# FABP3 Mediates Lipid Droplet Accumulation and Adhesive Capacity in Bovine Endometrial Epithelial Cells via PGE_2_/PTGER4/PPAR Axis

**DOI:** 10.3390/ani15233417

**Published:** 2025-11-26

**Authors:** Beibei Zhang, Yutong Yan, Ming Cheng, Tengfei Guo, Kangkang Gao, Aihua Wang, Pengfei Lin, Dong Zhou, Yaping Jin

**Affiliations:** 1 Key Laboratory of Animal Biotechnology of the Ministry of Agriculture, College of Veterinary Medicine, Northwest A&F University, Yangling 712100, China; 2021060265@nwafu.edu.cn (B.Z.); mingcheng984737409@163.com (M.C.); guotengfei@nwafu.edu.cn (T.G.); gkk171011@163.com (K.G.); wangaihua@nwafu.edu.cn (A.W.); linpengfei@nwafu.edu.cn (P.L.); 2 College of Animal Science and Technology, Fujian Vocational College of Agriculture, Fuzhou 350303, China; 3 College of Animal Sciences, Fujian Agriculture and Forestry University, Fuzhou 350002, China; yanyutong@fafu.edu.cn

**Keywords:** endometrial receptivity, estrous cycle, PGE_2_, FABP3, lipid droplet

## Abstract

Prostaglandin E_2_ (PGE_2_) has been implicated in multiple biological processes during pregnancy in ruminants. However, the regulatory effects of PGE_2_ on endometrial function during the diestrus period and its underlying molecular mechanisms remain poorly understood. Herein, PGE_2_ treatment promoted the accumulation of lipid droplets and induced cytoskeletal reorganization in bEECs. As a well-established inducer of lipid droplet formation, oleic acid (OA) treatment significantly increased the number of lipid droplets in bEECs, altered the distribution of F-actin and disrupted the expression patterns of key adhesion-related proteins. Transcriptomic analysis revealed that the PPAR signaling pathway was the key pathway that responded to PGE_2_ treatment in bEECs, and its downstream target gene FABP3 was markedly up-regulated. Knockdown of FABP3 led to a reduced number of BTC spheroids and down-regulation of adhesion-related proteins in bEECs while increasing the density of microvilli and up-regulating the expression of epithelial markers. Prostaglandin E receptor 4 (PTGER4) was the primary receptor that responded to PGE_2_ treatment, and PTGER4 knockdown or pharmacological inhibition with GW-627368 suppressed FABP3 expression in bEECs. Moreover, uterine samples from dairy cows at different stages of the estrous cycle showed that FABP3 expression was significantly elevated in the endometrium tissue during mid-diestrus compared to metestrus, with predominant localization in the luminal and superficial glandular epithelium. Collectively, these findings indicate that FABP3 regulates lipid droplet accumulation and adhesion ability in bEECs via the PGE_2_/PTGER4/PPAR signaling axis, providing new insights into the metabolic regulation of endometrial receptivity in ruminants.

## Simple Summary

To investigate the function of PGE_2_ on endometrial function during the diestrus period and its mechanisms, bovine endometrial epithelial cells (bEECs) were treated with P_4_ or P_4_ + PGE_2_. Results showed that PGE_2_ treatment promoted the accumulation of lipid droplets and induced cytoskeletal reorganization; the PPAR signaling pathway was the key pathway, and its downstream target gene FABP3 was markedly up-regulated; FABP3 knockdown reduced the adhesion of bovine trophoblast cell (BTC) spheroids to bEECs and down-regulation of adhesion-related proteins in bEECs while increasing the density of microvilli and up-regulated the expression of epithelial markers. These findings provide new insights into the metabolic regulation of endometrial receptivity in ruminants.

## 1. Introduction

Subfertility is a prevalent reproductive disorder in dairy cows, significantly compromising both productivity and economic returns [1,2]. Accumulating evidence indicates that prostaglandin (PG) levels in uterine lumen fluid and the expression of prostaglandin-related genes in the endometrium are closely linked to fertility [3,4]. As a key immunomodulatory and luteal protective factor in ruminants [5], PGE_2_ level in uterine lumen fluid increases rapidly during early pregnancy and comparable luteal phase [6], which is associated with conceptus elongation in dairy cows [3]. Notably, PGE_2_ predominantly exerts biological effects on the endometrium, owing to the high expression of its receptors (PTGER2 and PTGER4) in endometrial tissue and low expression level in the conceptus during the peri-implantation period of dairy heifers [6,7]. Meanwhile, PGs are important mediators of endometrial response to P_4_ and IFNT in the ovine uterus [8,9]. In ruminants, available data support that P_4_ from the ovary induces gene expression in the endometrium, which is further stimulated by factors (PGs, IFNT) to regulate the endometrial functions [1,10,11]. Despite these observations, the functional roles of PGE_2_ in endometrial physiology during the diestrus period, as well as the underlying molecular mechanisms, remain largely unclear.

Previous studies have demonstrated that the intrauterine infusion of meloxicam during mid-diestrus in dairy heifers inhibits the synthesis and secretion of prostaglandins, which leads to the alternations of lipids in uterine luminal fluid [12,13]. In ruminants, lipid droplets (LDs) in endometrium are considered a major source of lipids within the uterine fluid [14,15,16]. As a cytoplasmic organelle, LDs serve as a reservoir for energy storage, which plays regulatory roles in cellular signaling and metabolic processes [17]. The abundance of LDs in the endometrium exhibits dynamic fluctuations throughout the estrous cycle, exhibiting low levels during metestrus and increasing accumulation during diestrus, which corresponds with corpus luteum development in sheep and cattle [18,19,20]. The source of LDs is predominantly localized in the luminal and superficial glandular epithelia, with minor presence in stromal cells and deep glandular epithelium [20]. Progesterone is a primary inducer of LD accumulation, as demonstrated by progesterone supplementation experiments in ovariectomized ewes [19]. In contrast, administration of 17β-estradiol in the same experimental model results in a reduction in endometrial LD content [19]. However, the molecular mechanisms governing LD dynamics in endometrial epithelial cells during the estrous cycle and their functions in endometrial physiology remain to be elucidated.

Several genes related to lipid metabolism have been implicated in reproductive performance of dairy cows [3,20]. Fatty acid-binding protein 3 (FABP3), a critical member of the fatty acid-binding protein family, plays a central role in the intracellular transport and metabolism of long-chain fatty acids [21]. In dairy cows, FABP3 is highly expressed in endometrium and significantly elevated in pregnant cows compared to non-pregnant dairy cows [1,22], with peak expression on days 15 and 18 of gestation. Notably, FABP3 expression is higher in the endometrium of high-fertility heifers compared to those with subfertility [1]. Moreover, the abundance of FABP3 protein in uterine fluid decreases when the synthesis and secretion of uterine prostaglandins are inhibited in dairy heifers [13]. Collectively, these findings suggest that FABP3 may play a regulatory role in the function of uterine endometrium and potentially influence pregnancy outcomes in dairy cows.

This study aimed to investigate the effects of PGE_2_ on lipid droplet dynamics in endometrial epithelial cells, identify key responsive genes, and evaluate its functional roles in regulating endometrial cell function. The findings provide a theoretical basis for understanding the molecular mechanisms underlying endometrial receptivity and early embryonic development in ruminants.

## 2. Materials and Methods

### 2.1. Cell Culture and Treatment

In this study, bEECs were purchased from the American Type Culture Collection (ATCC^®^ CRL-2398™, Manassas, VA, USA) and cultured in DMEM/F-12 medium (HyClone, Logan, UT, USA) containing 10% fetal bovine serum (FBS, ZETA, New York, NY, USA) and incubated at 37 °C in a humidified 5% CO_2_ incubator. When bEECs reached 50–60% confluence, the medium was replaced with fresh phenol red-free DMEM/F-12 and 0.1% bovine serum albumin (BSA) for 12 h to synchronize cells. The bEECs were then cultured for an additional 24 h in phenol red-free DMEM/F-12 medium supplemented with 10% FBS and P_4_ (10^−7^ M; P0130, Sigma, Saint Louis, MO, USA) to mimic the uterine hormone environment of dairy cows during the luteal phase. Then, PGE_2_ (10^−8^–10^−6^ M; 14010, Cayman, Ann Arbor, MI, USA) was added to the medium, and the cells were cultured for 24 h. For inhibitor treatment, 5 and 10 μM PTGER2 inhibitor (TG-155; MedChemExpress, Newark, NJ, USA) or 5 and 10 μM PTGER4 inhibitor (GW627368; Selleck, Houston, TX, USA) were added to the medium 1 h prior to the PGE_2_ treatment.

For the oleic acid (OA) experiments, three experimental conditions were used. In the 5% FBS group (CON group), the medium was replaced with phenol red-free DMEM/F-12 containing 5% FBS. In the 1% BSA group, the medium was replaced with phenol red-free DMEM/F-12 containing 1% BSA to reduce the accumulation of intracellular lipid droplets. In the 5% FBS + OA group, the medium was replaced with phenol red-free DMEM/F-12 containing 5% FBS and 200 μM OA to promote lipid droplet accumulation.

### 2.2. Bulk RNA Sequencing (RNA-Seq)

Total RNA was extracted from bEECs after treatment with P_4_ or P_4_ + PGE_2_ using Trizol Reagent. Next, RNA was reverse-transcribed and amplified according to the manufacturer’s instructions. Sequencing libraries were constructed on the Illumina Novaseq 6000 platform (Genedenovo, Guangzhou, China). Briefly, Eukaryotic mRNA was enriched using Oligo(dT) beads. Then the enriched mRNA was fragmented into short fragments using fragmentation buffer and reverse-transcribed into cDNA with NEBNext Ultra RNA Library Prep Kit for Illumina (NEB, Ipswich, MA, USA). The purified double-stranded cDNA fragments were end-repaired, with a base added, and ligated to Illumina sequencing adapters. The ligation reaction was purified using the AMPure XP Beads (1.0×); then, the cDNA library was amplified by polymerase chain reaction (PCR). The resulting cDNA library was sequenced on the Illumina Novaseq6000. Firstly, we removed reads containing sequencing adapters or reads of poor quality in raw data. Secondly, transcriptional reads were mapped to the genome (Ensembl_release110) using HISAT2.2.4. With a threshold of absolute log_2_ fold change ≥0.58 and FDR < 0.05 [23,24], the differentially expressed genes (DEGs) were identified using DESeq2 (version 1.50.2). The RNA-seq analysis was performed by Genedenovo, using standard and consistent procedures. For functional analysis, KEGG and Gene set enrichment analysis (GSEA) were performed using the cluster Profiler R package (version 4.3.0).

### 2.3. Cell Transfection

The oligonucleotide sequences targeting bovine FABP3, PTGER2, PTGER4 mRNA and a scrambled sequence were designed using online software (https://rnaidesigner.thermofisher.com/rnaiexpress/ accessed on 24 October 2023). The sequences of shFABP3, shPTGER2, shPTGER4 and shN are shown in Supplementary Table S1. The virus packaging and cell transfection were performed as previously described [25,26]. These shRNA fragments were cloned into the pCD513B-U6 vector using *EcoR* I and *BamH* I restriction enzymes. After screening with ampicillin, the correct vectors were co-transfected with the third-generation lentivirus package plasmids (encoding GAG, REV, and VSV-G protein) into HEK293 cells with TurboFect Transfection Reagent (R0533, Invitrogen, Carlsbad, CA, USA). The supernatant was collected and filtered using a 0.45 μm PVDF filter after transfection for 48 h and then used to infect bEECs. Cells with stable gene knockdown were selected with 0.2 μg/mL puromycin.

The cDNA encoding FABP3 was amplified and cloned into the lenti-CMV-MCS-flag-IRES-PUPO vector using *Nhe I* and *Xho I* restriction enzymes. The sequences of primers are shown in Supplementary Table S1. After screening with ampicillin, the correct vector was co-transfected with the second-generation lentivirus package plasmids (encoding GAG, Pol, REV, Tat and VSV-G protein) into HEK293 cells with TurboFect Transfection Reagent (R0533, Invitrogen). The supernatant was collected and filtered with a 0.45-μm PVDF filter after transfection for 48 h and used to infect bEECs. Cells with stable gene overexpression were selected with 0.2 μg/mL puromycin. Transfection with an empty vector was used as a control group.

### 2.4. Tissue Collection

Uteri from healthy cows, ipsilateral to the ovary containing a corpus luteum (CL), were immediately collected at a commercial slaughterhouse under veterinary supervision. The stage of the estrous cycle was determined according to the morphological observation of ovaries (follicles and CL) and uterus, following previous reports [27,28]. Endometrial tissues were obtained from dairy cows in metestrus (days 2–5) and diestrus (days 11–15) (*n* = 3 per stage) [29]. The endometrium was immediately separated from the harvested uterus and snap-frozen in liquid nitrogen, transported to the laboratory and stored at −80 °C.

### 2.5. Progesterone Determination

To confirm the accuracy of the estrous cycle, progesterone (P_4_) concentration in endometrial tissues was measured according to the report [29]. The concentration of P_4_ extracted from endometrial tissues using petroleum ether was measured using an Enzyme-Linked Immunosorbent Assay (ELISA) (002401, MEIMIAN, Yancheng, China) following the manufacturer’s instructions. The standard curve ranged from 0.1 to 100 ng/mL, with a sensitivity of 0.15 ng/mL. The intra- and inter-assay coefficients of variation were 8.7% and 11%, respectively.

### 2.6. Immunohistochemistry

Paraffin-embedded uterine tissues were deparaffinized, rehydrated, and then stained with the streptavidin–peroxidase method following the previous report [30]. Briefly, antigen retrieval was performed by boiling the sections in citrate antigen retrieval solution (Solarbio, Beijing, China) for 20 min. Subsequently, the universal SP staining kit (Maixin Biotechnologies, Fuzhou, China) was used according to the manufacturer’s instructions. After blocking, the sections were incubated overnight at 4 °C with primary antibodies, including anti-PGR antibody (1:200, 25871-1-AP, Proteintech, Wuhan, China) [31], anti-FABP3 antibody (1:200, 10676-1-AP, Proteintech) [32] and a negative control (1:1000, IgG, Abcam, Cambridge, UK). The sections were subsequently incubated with the second antibody at 37 °C for 2 h and incubated with streptavidin–biotin peroxidase at 25 °C for 40 min after washing three times with PBS. Visualization was performed using diaminobenzidine (DAB) as the chromogen, followed by light counterstaining with hematoxylin. Images were captured using a microscope (Nikon, Tokyo, Japan).

### 2.7. RNA Extraction and Real-Time Quantitative PCR

Total RNA was extracted from the bEECs using TRIzol reagent (TaKaRa, Tokyo, Japan) according to the manufacturer’s protocols. The RNA (1 μg) was converted into complementary DNA (cDNA) using a PrimeScript™ RT reagent kit with gDNA Eraser (TaKaRa). Real-time quantitative PCR (qPCR) was performed using SYBR Green Master Mix (Vazyme, Najing, China) in a Bio-Rad CFX96 (Bio-Rad, Hercules, CA, USA), according to the manufacturer’s protocol. Gene expression of mRNA was normalized to the *RPS9* gene. Relative expression levels were calculated with the 2^−∆∆ct^ method. The primer sequences are shown in Supplementary Table S2.

### 2.8. Western Blot

The samples were lysed with RIPA buffer (Solarbio, Beijing, China), according to the manufacturer’s protocol. The total protein concentration was measured using the BCA assay (Keygen Biotech, Nanjing, China). The total proteins (20 μg) were loaded onto an SDS-PAGE gel and then transferred to PVDF membranes (Millipore, Bedford, MA, USA). After blocking with 5% non-fat milk for 2 h, the membranes were incubated with primary antibodies at 4 °C overnight, including anti-PLIN2 antibody (1:1000, ab181452, Abcam) [33], anti-CDH1 antibody (1:1000, 3195, Cell Signaling Technology, Danvers, MA, USA) [34], anti-ZO-1 antibody (1:1000, 21773-1-AP, Proteintech, bovine), anti-β-catenin antibody (1:500, sc-53484, Santa Cruz, CA, USA) [35], anti-FABP3 antibody (1:1000, ab133585, Abcam), anti-OPN antibody (1:1000, 22952-1-AP, Proteintech) [36], anti-ITGB3 antibody (1:1000, CY5237, Abways, Shanghai, China), anti-PTGER2 antibody (1:1000, ab167171, Abcam), anti-PTGER4 antibody (1:1000, ab217966, Abcam), anti-β-Tubulin antibody (1:2000, 56739S, Cell signaling Technology) [37] and anti-GAPDH (1:2000, 60004-1-Ig, Proteintech) [38]. Subsequently, the membranes were incubated for 1 h with an HRP-labeled secondary antibody at room temperature.

### 2.9. Measurement of Cell Viability

The bEECs (5 × 10^3^ cells/well) were seeded into a 96-well plate. Following 36 h of inhibitor treatment, cells were incubated with 10 μL reagent of the Cell Counting Kit-8 (C0037, Beyotime, Shanghai, China) for 2 h at 37 °C. The OD value was measured at 450 nm using a Multimode Microplate Reader (TECAN, Männedorf, Switzerland).

### 2.10. Cell Proliferation Assay

The bEECs with stable FABP3 knockdown or overexpression were harvested using trypsin after washing once with PBS. Cells were then centrifuged at 2000 rpm for 5 min and subsequently fixed in cold 70% ethanol overnight at 4 °C. After fixation, samples were stained using the Cell Cycle Detection Kit (KGA9101, KeyGEN biotech, Co., Ltd., Nanjing, China) according to the manufacturer’s instructions. DNA content was measured using a BD FACSAria II SORP flow cytometer (Franklin Lakes, NJ, USA), and the number of cells in the G_1_, S, and G_2_ phases was calculated using Modfit 3.1 software.

The bEECs with FABP3 knockdown or overexpression were cultured in 24-well plate at a density of 5 × 10^4^ cells/well and incubated with 10 μM EdU (C0075S, Beyotime) for 6 h. After removing the medium and washing, cells were permeabilized with PBS containing 3% Triton X-100. Subsequently, staining was performed according to the manufacturer’s instructions. Samples were observed by fluorescence microscope (Nikon).

### 2.11. Immunofluorescent Staining

The cell samples were fixed with 4% formaldehyde for 30 min, rinsed three times with PBS, and permeabilized with PBST (0.1% Triton X-100 in PBS) for 5 min. After blocking with 3% BSA for 1 h and washing three times, the samples were incubated with anti-FABP3 antibody (1:500, ab133585, Abcam) overnight at 4 °C; after washing three times with PBS, incubation with Alexa-labeled donkey anti-rabbit IgG (1:1000, A16028, Invitrogen) was performed at room temperature for 1 h. The nuclei were counterstained with 4′,6-diamidino-2-phenylindole (DAPI) (C1002, Beyotime) for 5 min, and the cells were observed using laser-scanning confocal microscopy (Nikon, Tokyo, Japan).

### 2.12. Scanning Electron Microscope

The cell samples were fixed in a 3% glutaraldehyde-buffered solution for 30 min at room temperature. Afterward, the samples were rinsed three times with PBS and subsequently fixed in a 1% rhodium acid solution for 1 h at 4 °C. Following rinsing in demineralized water, the samples were dehydrated through a graded ethanol series up to 100% ethanol. They were then critical point-dried using liquid CO_2_ (Leica EM CPD 300 Manual) and sputter-coated with a 7 nm gold/palladium layer (Leica EM ACE 200). All samples were examined using a JSM-IT700HR scanning electron microscope (JEOL Ltd., Tokyo, Japan).

### 2.13. In Vitro Implantation Assay

The BTCs were a gift from associate professor Xiangguo Wang of Beijing University of Agriculture [39]. In this study, BTCs were cultured in DMEM/F12 medium containing 10% FBS and incubated at 37 °C in a humidified 5% CO_2_ incubator. The in vitro embryo implantation was simulated using a spheroid coculture assay, performed according to the previous study [30]. The cell suspension containing approximately 1.0 × 10^6^ cells was stained with CellTracker Red CMFDA (0.5 μM; Yeasen Biotech, Shanghai, China) and cultured on an orbital shaker rotating at 200 rpm for 10 h at 37 °C. The spheroids had a diameter of approximately 100–200 μm and contained about 200–1000 cells each. Then, the BTC spheroids were delivered onto monolayer bEECs after PGE_2_ treatment. The bEECs and BTC spheroids (approximately 100 spheroids per well) were cocultured for 2 h at 37 °C. After washing three times with PBS, the BTC spheroids were counted under a fluorescence microscope. The attachment rate is expressed as the percentage of seeded spheroids.

### 2.14. BODIPY Staining

The cell samples were fixed with 4% formaldehyde for 30 min, rinsed three times with PBS, each time for 5 min, and permeabilized by incubation with PBST (0.1% Triton X-100 in PBS) for 5 min. After washing three times with PBS, incubation with Alexa Fluor^TM^ 594 phalloidin (A12381, Invitrogen) was performed at room temperature for 20 min. Then, the samples were incubated with 10 μg/mL BODIPY 493/503 (D3922, Invitrogen) for 30 min at room temperature after washing three times. Cell nuclei were counterstained with DAPI for 5 min, and the cells were observed using laser-scanning confocal microscopy (Nikon).

The cell samples were washed with precooled PBS and harvested with trypsin. Next, the cells were centrifuged at 2000 rpm for 5 min and then stained using 10 μg/mL BODIPY 493/503 for 30 min. After washing three times, the fluorescence intensity of samples was tested by flow cytometry. The fluorescence intensity was analyzed by Image Pro Plus 6.0 software.

### 2.15. Statistical Analysis

Results were presented as the means ± SEM and obtained from at least 3 independent experiments. Statistical analyses were performed with GraphPad Prism software (version 8.0) using unpaired Student’s *t*-test for experiments with two groups or one-way analysis of variance (ANOVA) followed by Tukey’s post hoc test between multiple groups. The results with statistically significant differences are indicated by asterisks (*p* < 0.05 denoted by *, *p* < 0.01 denoted by **, *p* > 0.05 denoted by ns).

## 3. Results

### 3.1. PGE_2_ Treatment Induces Lipid Droplet Accumulation and Alternation of Cell Morphology in Bovine Endometrial Epithelial Cells

**Figure 1:**
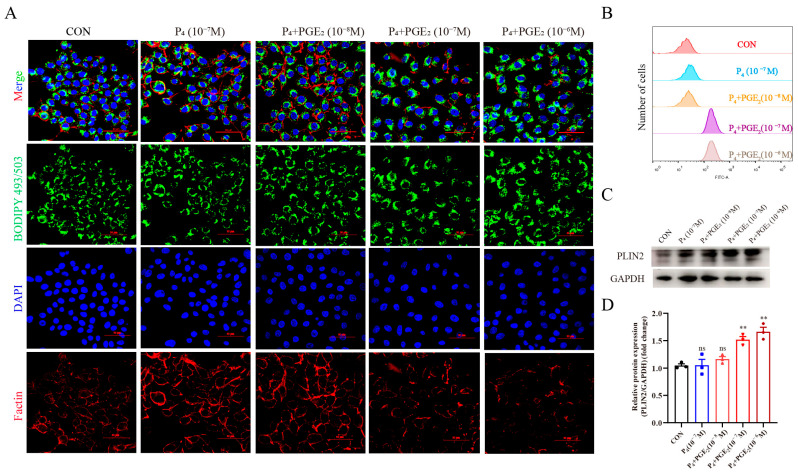
PGE_2_ treatment induces lipid droplet accumulation and alternation of cell morphology of bEECs: (**A**) The representative images of lipid droplets and cytoskeleton staining in bEECs after treatment with P_4_ and/or PGE_2_. BODIPY 493/503 (green), F-action (red) and DAPI (blue) marked lipid droplets, cytoskeleton and cell nuclei, respectively. Scale bar: 50 μm. (**B**) The fluorescence intensity analysis of BODIPY 493/503 staining in bEECs after treatment with P_4_ and/or PGE_2_. Y-axis represents the number of cells detected at each fluorescence. (**C**,**D**) The representative images and analysis of PLIN2 expression in bEECs after treatment with P_4_ and/or PGE_2_, respectively. The data are means ± SEM of three independent experiments. Statistically significant differences are indicated by asterisks: ** *p* < 0.01, ns *p* > 0.05.

To explore the potential role of PGE_2_ in endometrium function during diestrus, P_4_ and PGE_2_ were used to mimic hormonal changes, and detect lipid droplet accumulation in bEECs after treatment. Compared with the CON group, P_4_ treatment had a limited effect on lipid droplet accumulation, while the combination of P_4_ and PGE_2_ treatment significantly promoted lipid droplet accumulation (Figure 1A). The fluorescence intensity of BODIPY 493/503 was analyzed by flow cytometry, and the results showed that PGE_2_ treatment promoted lipid droplet accumulation (Figure 1B). Moreover, PGE_2_ treatment significantly increased PLIN2 expression as a marker of lipid droplet (Figure 1C,D). Meanwhile, P_4_ + PGE_2_ treatment led to alterations in cell morphology, with partial cytoskeleton disorganization (F-actin). Since the 10^−7^ M PGE_2_ concentration enhanced PLIN2 expression, this concentration was used for subsequent processing treatment and analysis. The above results indicated that PGE_2_ treatment induced lipid droplet accumulation and alternation of cell morphology in bEECs.

### 3.2. The Number of Lipid Droplets Is Related to Cell Morphology in Bovine Endometrial Epithelial Cells

**Figure 2:**
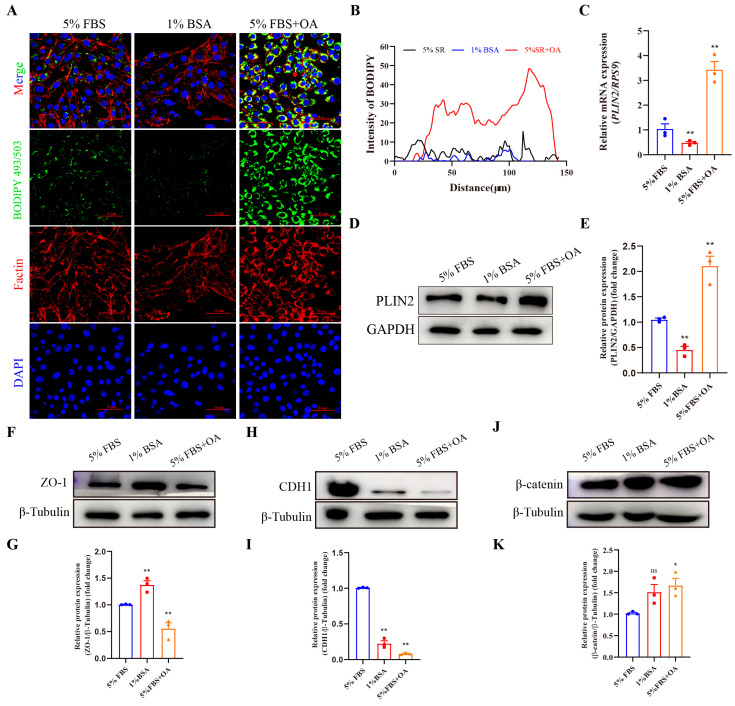
The number of lipid droplets in bEECs is related to the cell morphology: (**A**) The representative images of lipid droplets and cytoskeleton staining in bEECs after treatment with 5% FBS, 1% BSA or 5% FBS + OA. BODIPY 493/503, F-action and DAPI marked lipid droplets, cytoskeleton and cell nuclei, respectively. Scale bar: 50 μm. (**B**) The fluorescence intensity analysis of BODIPY 493/503 staining in bEECs after treatment with 5% FBS, 1% BSA or 5% FBS + OA. (**C**) The analysis of *PLIN2* expression in bEECs after treatment with 5% FBS, 1% BSA or 5% FBS + OA. (**D**,**E**) The representative images and analysis of PLIN2 expression in bEECs after treatment with 5% FBS, 1% BSA or 5% FBS + OA, respectively. (**F**,**G**) The representative images and analysis of ZO-1 expression in bEECs after treatment with 5% FBS, 1% BSA or 5% FBS + OA, respectively. (**H**,**I**) The representative images and analysis of CDH1 expression in bEECs after treatment with 5% FBS, 1% BSA or 5% FBS + OA, respectively. (**J**,**K**) The representative images and analysis of β-catenin expression in bEECs after treatment with 5% FBS, 1% BSA or 5% FBS + OA, respectively. The data are means ± SEM of three independent experiments. Statistically significant differences are indicated by asterisks: * *p* < 0.05, ** *p* < 0.01, ns *p* > 0.05.

Previous studies show that there is a correlation between cytoskeletal remodeling and lipid droplets [40,41]. Hence, the lipid droplet levels of bEECs were detected under different culture conditions in this study. The content of lipid droplets in bEECs was reduced when cultured in serum-free medium added 1% BSA, while the number of lipid droplets was increased when cultured in medium added 200 μM oleic acid (OA). Cell morphology was observed by staining F-actin, a cytoskeletal component, with phalloidin. Compared with the control group (5% FBS), the cells in the 1% BSA group were flattened and closely arranged (Figure 2A,B), and PLIN2 expression was significantly down-regulated in the 1% BSA group (Figure 2C–E). After treatment with 5% FBS + OA, the cells were rounder in shape and loosely arranged (Figure 2A,B), and PLIN2 expression was significantly up-regulated compared with 5% FBS group (Figure 2C–E).

Epithelial cell tight junction protein (ZO-1) and adherens junction proteins (CDH1, β-catenin) were detected (Figure 2F–K). Results showed that alternations in cellular lipid metabolism significantly reduced CDH1 expression (Figure 2H,I). Compared with the control group (5% FBS), the protein level of ZO-1 was significantly up-regulated in the 1% BSA group but markedly decreased in the 5% FBS + OA group (Figure 2F,G). The protein level of β-catenin showed no significant changes in the 1% BSA group, while it was down-regulated in the 5% FBS + OA group (Figure 2J,K). Collectively, the number of lipid droplets was related to cell morphology in bEECs, and disruption of lipid droplets altered cell morphology.

### 3.3. RNA-Seq Reveals PPAR Signaling Pathway and Its Key Molecules Respond to PGE_2_ Treatment in Bovine Endometrial Epithelial Cells

**Figure 3:**
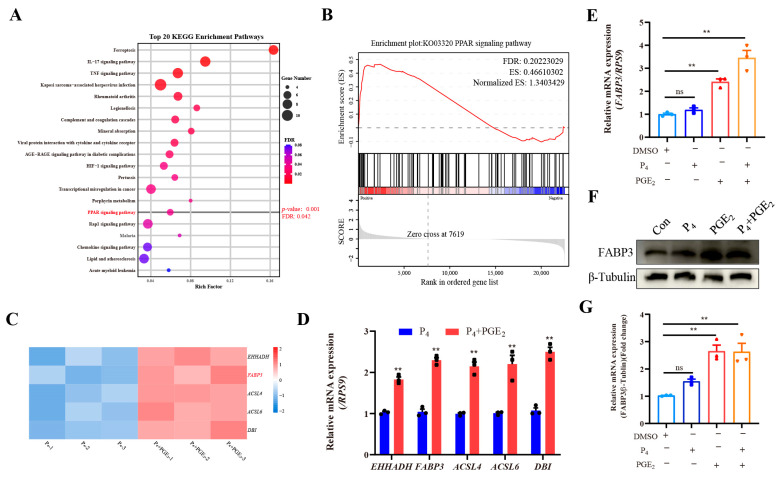
PGE_2_ treatment promotes PPAR signaling pathway and FABP3 expression in bEECs: (**A**) The top 20 pathways from KEGG analysis between P_4_ and P_4_ + PGE_2_ groups. (**B**) The GSEA of PPAR signaling pathway between P_4_ and P_4_ + PGE_2_ groups. (**C**) The heatmap of key gene (*EHHADH*, *FABP3*, *ACSL4*, *ACSL6* and *DBI*) expression related to PPAR signaling pathway. (**D**) The analysis of key gene (*EHHADH*, *FABP3*, *ACSL4*, *ACSL6* and *DBI*) expression in bEECs between P_4_ and P_4_ + PGE_2_ groups. (**E**) The analysis of *FABP3* expression in bEECs after P_4_ and/or PGE_2_ treatment. (**F**,**G**) The representative images and analysis of FABP3 in bEECs after P_4_ and/or PGE_2_ treatment. The data are means ± SEM of three independent experiments. Statistically significant differences are indicated by asterisks: ** *p* < 0.01, ns *p* > 0.05.

To analyze the signaling response to PGE_2_ treatment in bovine endometrial epithelial cells, cells from the P_4_ and P_4_ + PGE_2_ treatment groups were collected and subjected to RNA-seq. The principal component analysis (PCA) is shown in Figure S1A, and a total of 230 DEGs were identified between the P_4_ and P_4_ + PGE_2_ groups (Figure S1B). The volcano plot and heatmap of DEGs are shown in Figure S1C and S1D, respectively, and these DEGs were used for GO analyses and are shown in Figure S1E. The KEGG analysis of these DEGs is shown in Figure 3A, showing that PGE_2_ treatment significantly enriched Ferroptosis, IL-17 signaling pathway, TNF signaling pathway and others. Notably, the PPAR signaling pathway (*p* = 0.001 and FDR = 0.042) was enriched after PGE_2_ treatment (Figure 3A), which was associated with lipid droplet accumulation. The GSEA result showed that PGE_2_ treatment enhanced the PPAR signaling pathway (Figure 3B). The mRNA expression of key genes (EHHADH, FABP3, ACSL4, ACSL6 and DBI) was significantly up-regulated after PGE_2_ treatment (Figure 3C), and these alternations were further confirmed by q-PCR results (Figure 3D).

According to the above analysis, FABP3 may be the key gene that responds to PGE_2_ treatment in bEECs and regulates lipid droplet accumulation. Therefore, we further analyzed FABP3 expression after P_4_ and/or PGE_2_ treatment. The results of q-PCR and WB showed that P_4_ treatment had a limited effect on FABP3 expression; PGE_2_ treatment alone significantly increased the mRNA and protein expression of FABP3; and the combination treatment further enhanced this trend (Figure 3E–G). Collectively, the PPAR signaling pathway and FABP3 were the key molecules responding to PGE_2_ treatment in bovine endometrial epithelial cells.

### 3.4. FABP3 Regulates the Proliferation of Bovine Endometrial Epithelial Cells

To explore the function of FABP3, stable knockdown or overexpression of FABP3 in bEECs was established. The efficiency of FABP3 knockdown or overexpression was verified by RT-qPCR, WB and IF (Figure S2). The results showed that shFABP3-2 and shFABP3-3 constructs had high interference efficiency and were used for subsequent experiments (Figure S2A–C). The results of IF staining showed that FABP3 was mainly located in the cytoplasm (Figure S2D). Compared with the CON group, FABP3 expression was significantly increased (Figure S2E) in FABP3 overexpression (FABP3 OE) group. The FLAG tag was inserted during the construction of the lentiviral overexpression vector, so the FLAG tag was expressed simultaneously with FABP3 (Figure S2E–H).

**Figure 4:**
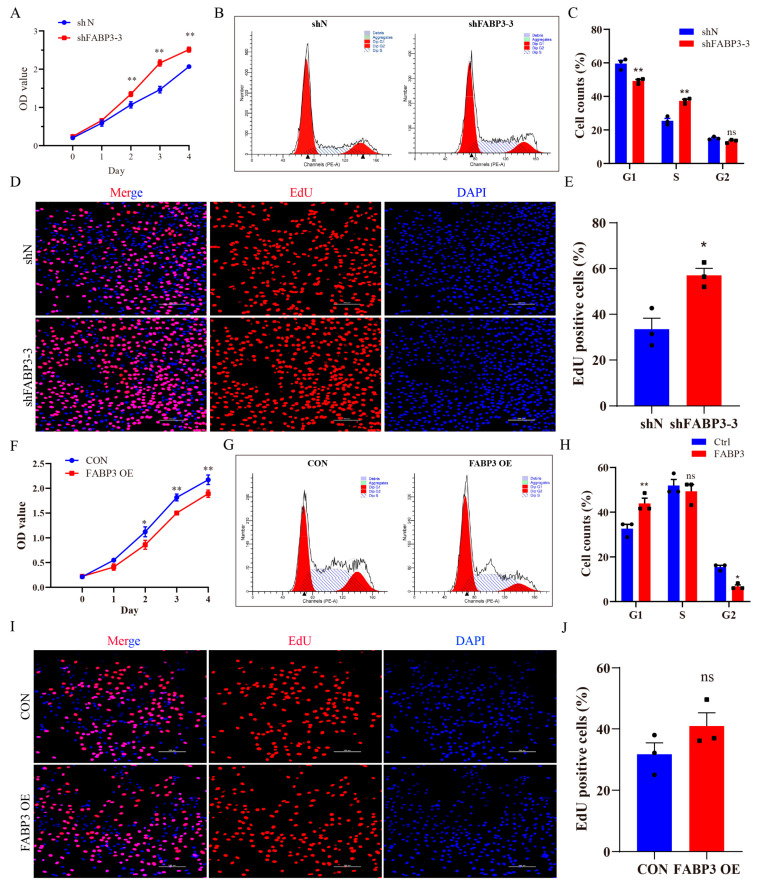
FABP3 expression regulates the cell proliferation and cycle in bEECs: (**A**) The analysis of cell proliferation in bEECs after FABP3 interference by CCK8. (**B**,**C**) The detection and analysis of cell cycle in bEECs after FABP3 interference by flow cytometer. (**D**,**E**) The representative images and analysis of EdU staining in bEECs between shN and shFABP3-3 groups. Scale bar: 100 μm. (**F**) The analysis of cell proliferation in bEECs after FABP3 overexpression by CCK8. (**G**,**H**) The detection and analysis of cell cycle in bEECs after FABP3 overexpression with by flow cytometer. (**I**,**J**) The representative images and analysis of EdU staining in bEECs between CON and FABP3-overexpression groups. Edu (red), DAPI (blue), Scale bar: 100 μm. The data are means ± SEM of three independent experiments. Statistically significant differences are indicated by asterisks: * *p* < 0.05, ** *p* < 0.01, ns *p* > 0.05.

To study the function of FABP3 in bEECs, the effect of FABP3 on cell proliferation was first detected. The results of CCK8 indicated that the knockdown or overexpression of FABP3 significantly altered the cell cycle (Figure 4A,F). Concretely, the knockdown of FABP3 increased the proportion of cells in S phase and decreased the cell proportion in G1 phase, while there was no significant difference in G2 phase (Figure 4B,C). The EdU staining results showed that the proportion of positive cells in the shFABP3-3 group was significantly greater than in the shN group (Figure 4D,E). In contrast, after overexpression of FABP3, the proportion of cells in the G1 phase increased (Figure 4G,H), the proportion of cells in the G2 phase decreased (Figure 4G,H), and there was no significant change in cells in the S phase (Figure 4G,H). Moreover, the EdU staining results showed that there was no significant difference in the proportion of positive cells between the CON and FABP3 overexpression groups (Figure 4I,J). The above results showed that FABP3 regulated the proliferation of bEECs, and disruption of FABP3 expression altered the cell cycle.

### 3.5. FABP3 Regulates the Adhesion Ability of Bovine Endometrial Epithelial Cells

**Figure 5:**
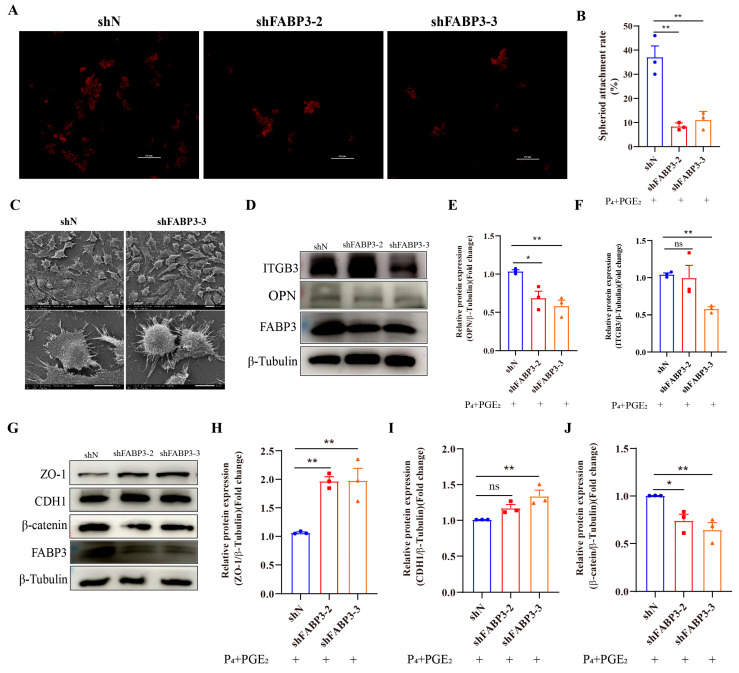
The expression of FABP3 regulates endometrial epithelial cell remodeling: (**A**,**B**) The representative images and analysis of BTCs spheroid adhesion rate in bEECs after FABP3 knockdown, respectively. BTCs: Bovine placental trophoblast cells. Scale bar: 200 μm. (**C**) Representative images of scanning electron microscope in the microvilli structure on the surface of bEECs after FABP3 knockdown. Scale bar: 20 μm (Others), 10 μm (Enlarge). (**D**–**F**) The representative images and analysis of the protein expression related to cell adhesion (OPN and ITGB3) in bEECs between shN and shFABP3 groups, respectively. (**G**–**J**) The representative images and analysis of the protein expression (ZO-1, CDH1 and β-catenin) in bEECs between shN and shFABP3 groups, respectively. The data are means ± SEM of three independent experiments. Statistically significant differences are indicated by asterisks: * *p* < 0.05, ** *p* < 0.01, ns *p* > 0.05.

To further understand the impact of FABP3 on the dynamic remodeling of endometrium, we detected the effects of FABP3 interference or overexpression in bEECs on cell adhesion ability, the number of microvilli and the related protein expression. The attachment of BTC spheroids in the shFABP3 group was lower than that in the shN group (Figure 5A,B). The adhesion ability of endometrial epithelial cells is related to the number of surface microvilli. The results of scanning electron microscopy further indicated that FABP3 interference led to an increase in microvilli on the surface of bEECs (Figure 5C). The expression of adhesion-related proteins (OPN and ITGB3) was significantly decreased in the shFABP3 group compared to the shN group (Figure 5D–F). Interference with FABP3 in bEECs led to the up-regulation of ZO-1 (Figure 5G,H) and CDH1 (Figure 5G,I) while down-regulating β-catenin expression (Figure 5G,J). Nevertheless, overexpression of FABP3 in bEECs had no significant effect on the adhesion ability (Figure S3A,B) and the microvilli on the surface (Figure S3C). The expression of adhesion-related proteins (OPN and ITGB3) did not change significantly (Figure S3D–F). However, the expression level of cell remodeling-related proteins (ZO-1 and CDH1) was significantly decreased in the FABP3 overexpression group compared with the CON group (Figure S3G–I). Additionally, the expression level of β-catenin protein was significantly decreased (Figure S3G,J). Collectively, FABP3 was involved in regulating the adhesion ability of bEECs.

### 3.6. PGE_2_ Binds to PTGER4 to Regulate FABP3 Expression of Bovine Endometrial Epithelial Cells

**Figure 6:**
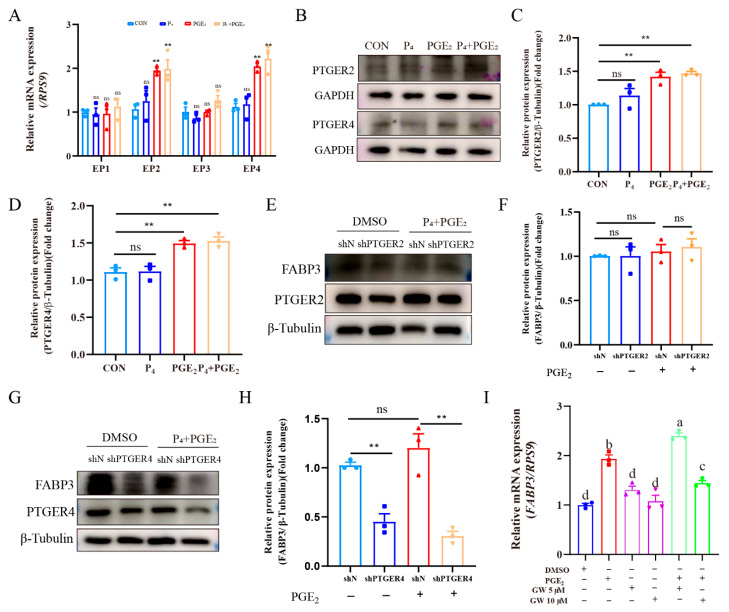
PGE_2_ binds to PTGER4 to regulate FABP3 expression in bEECs: (**A**) The difference in PTGER (*PTGER1*, *PTGER2*, *PTGER3* and *PTGER4*) expression in bEECs after treatment with P_4_ and/or PGE_2_. (**B**–**D**) The representative images and analysis of PTGER2 and PTGER4 expression in bEECs after treatment with P_4_ and/or PGE_2_. (**E**,**F**) The representative images and analysis of PTGER2 expression in bEECs between shN and shPTGER2 groups after treatment with DMSO or P_4_ + PGE_2_. (**G**,**H**) The representative images and analysis of PTGER4 expression in bEECs between shN and shPTGER4 groups after treatment with DMSO or P_4_ + PGE_2_. (**I**) The analysis of FABP3 expression in bEECs after treatment with DMSO, PGE_2_ or the inhibitor of PTGER4 (GW627368). The data are means ± SEM of three independent experiments. Statistically significant differences are indicated by asterisks: ** *p* < 0.01, ns *p* > 0.05. Bars with different letters are significantly different (*p* < 0.05).

The PGE_2_ mainly exerts its biological functions through its receptors, so we detected the expression of PTGERs. Results showed that PGE_2_ treatment significantly up-regulated *PTGER2* and *PTGER4* expression of bEECs (Figure 6A), and the combination treatment of P_4_ and PGE_2_ also significantly up-regulated *PTGER2* and *PTGER4* expression of bEECs (Figure 6A). The WB results further confirmed the up-regulation of PTGER2 and PTGER4 expression (Figure 6B–D). Subsequently, stable knockdown of PTGER2 and PTGER4 was constructed in bEECs (Figure S4A–D). Results showed that knockdown of PTGER2 has no significant effect on FABP3 expression after treatment (Figure 6E,F), while knockdown of PTGER4 significantly inhibited FABP3 expression after treatment (Figure 6G,H). Moreover, PTGER2 (TG-155) and PTGER4 (GW-627368) inhibitors were used to treat bEECs. TG-155 or GW-627368 (5 μM) treatment had no significant effect on cell viability (Figure S4E,F) and *FABP3* expression (Figure S4G). However, 10 μM GW-627368 treatment inhibited the up-regulation of *FABP3* by PGE_2_ treatment (Figure 6I). Collectively, PGE_2_ mainly binds to PTGER4 to regulate FABP3 expression in bEECs.

### 3.7. Immunolocalization and Expression of FABP3 in the Endometrial Tissue of Dairy Cows at Different Stages of Estrus Cycle

**Figure 7:**
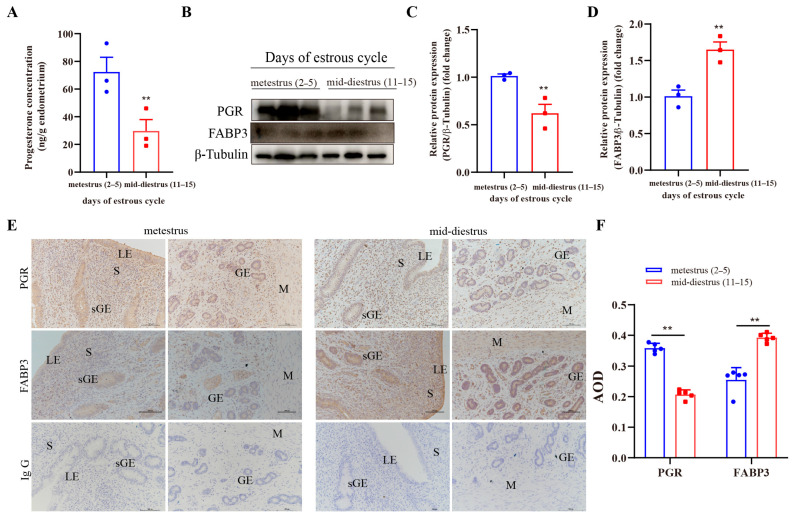
The expression of PGR and FABP3 in endometrial tissues of dairy cows at different stages of the estrus cycle: (**A**) The analysis of progesterone concentration in endometrial tissues of dairy cows at different stages of the estrus cycle. (**B**–**D**) The representative images and analysis of PGR and FABP3 expression in endometrial tissues of dairy cows at different stages of the estrus cycle, respectively. (**E**,**F**) The representative images and quantitative analysis of PGR and FABP3 staining in endometrial tissues of dairy cows at different stages of the estrus cycle, respectively. LEs: luminal epithelial cells; sGE: superficial glandular epithelium; GEs: glandular epithelial cells; S: stromal cells; M: myometrium. Scale bar = 100 μm. AOD = Average Optical Density. The data are means ± SEM of three independent experiments. Statistically significant differences are indicated by asterisks: ** *p* < 0.01.

To determine FABP3 expression in the endometrium of dairy cows at different stages of the estrous cycle, uterine samples were collected during metestrus (days 2–5) and mid-diestrus (days 11–15). The uterine tissue samples were further screened for P_4_ content and PGR expression in the endometrial tissues to confirm the accuracy of the estrous cycle stages. Compared to the tissues in the metestrus phase, both progesterone levels and PGR expression were reduced in the endometrium during the mid-diestrus period (Figure 7A–C), while FABP3 expression in endometrial tissue was elevated in mid-diestrus phase (Figure 7D). Moreover, FABP3 was mainly expressed in endometrial LE and sGE, while it was expressed at low levels in the deeper glandular epithelial cells and the myometrium. Its abundance was increased in the endometrium during mid-diestrus compared to the metestrus (Figure 7E,F). Collectively, FABP3 was elevated in the endometrial tissue of dairy cows during mid-diestrus compared to metestrus, with predominant localization in the luminal and superficial glandular epithelium.

## 4. Discussion

Previous studies find that the number of lipid droplets in the endometrial epithelial cells of cattle [20,42] and sheep [43] significantly varies at different stages of the estrous cycle. The number of lipid droplets in the endometrial epithelium is relatively low during the metestrus period and gradually increases during diestrus [16]. The content of PGE_2_ in the uterine lumen fluid of dairy cows also rapidly increases during the diestrus and pre-implantation period [3,6]. During the diestrus in dairy heifers, the inhibition of prostaglandin synthesis and secretion in the endometrium affects lipid content in uterine fluid [12,13]. In this study, PGE_2_ treatment promoted the accumulation of lipid droplets in bEECs after P_4_ pre-treatment. These results suggest that PGE_2_ collaborates with P_4_ to regulate the accumulation of lipid droplets in endometrium.

In ruminants, dynamic remodeling of endometrium is an essential prerequisite for successful embryonic implantation and represents a pivotal factor influencing the differential fertility in dairy cows [1], involving morphological changes in endometrial epithelial cells. The remodeling of junctional complexes and membrane-associated cytoskeleton is vital for epithelial transformation [44,45]. In dairy cows, the accumulation of endometrial lipid droplets during the luteal phase aligns with the timing of dynamic endometrial remodeling. In this study, the accumulation of lipid droplets promoted the remodeling of cytoskeleton (F-actin) by adjusting the culture conditions of bEECs; the expression of tight junction protein (ZO-1) and epithelial marker protein E-cadherin (CDH1) decreased, while it increased the expression of β-catenin protein. In contrast, reducing lipid droplet content by serum starvation led to the up-regulation of ZO-1 expression. Although serum starvation effectively reduced lipid droplets in cells, it has limitations, including a decrease in cell viability. As a key component of the tight junction, ZO-1 establishes a crucial connection between the transmembrane protein occludin and actin cytoskeleton [46,47]. The CDH1 engages with intracellular β-catenin (CTNNB1) and anchors the complex to the actin cytoskeleton [48]. This association is critical for preserving intercellular adhesion, and the onset of embryo implantation is marked by dynamic alterations of the expression and localization of these proteins [49,50]. These results further indicate that the dynamic changes in lipid droplets in endometrial epithelial cells are closely related to cell remodeling.

Herein, the RNA-seq analysis showed that the PPAR signaling pathway, which is involved in lipid metabolism in endometrial epithelial cells, was activated after PGE_2_ treatment. Peroxisome proliferator-activated receptors (PPARs) are members of the nuclear hormone receptor superfamily, including PPARα, PPARβ/δ, and PPARγ [51]. They form a specific heterodimer complex with retinoid X receptor (RXR) and bind to a specific DNA sequence located in the promoter region of target genes, known as PPAR response elements (PPREs) [52]. PPARs are regarded as important regulators of lipid metabolism [53,54], and the PPAR signaling pathway plays a significant role in placental formation, embryonic development and endometrial receptivity formation in mammals [51,55,56]. Previous studies find that PPARδ plays a significant role in regulating the formation and transport of lipid droplets in goat mammary epithelial cells, and FABP3 is the core gene downstream of PPARδ [3,57]. This study found that PGE_2_ treatment significantly induced FABP3 expression in endometrial epithelial cells under P_4_ pretreatment. In ruminants, many genes related to pregnancy in the endometrium firstly require the stimulation of progesterone and are induced by other signaling molecules [10,58]. These findings suggest that FABP3 expression may be involved in P_4_ induction and PGE_2_ stimulation in endometrial epithelial cells.

Consistent with the existing research [1,3,22], FABP3 is highly expressed in the endometrium during mid-diestrus in dairy cows and is mainly expressed in the endometrial luminal epithelium and superficial glandular epithelium. The expression of FABP3 varies in the endometrium of dairy cows with different fertility levels [1]. Moreover, FABP3 is highly expressed in the endometrium of dairy cows with high fertility, suggesting that it plays a role in regulating the functional changes in endometrium during early pregnancy [1]. This study found that FABP3 knockdown in bEECs increased the number of microvilli, reduced the number of trophoblast cell spheres, and decreased the expression of adhesion proteins (ITGB3 and OPN). However, there was a limited effect of FABP3 overexpression on cell adhesion and microvilli, which may be attributed to compensatory mechanisms activated in response to FABP3 modulation. Previous studies have shown that FABP3 overexpression is oncogenic [59]. Cancer cells undergo the epithelial-to-mesenchymal transition (EMT) process, resulting in a decrease in the expression of ZO-1 and CDH1 [60]. In this study, overexpression of FABP3 also resulted in a decrease in the levels of ZO-1 and CDH1, suggesting that excessive exogenous FABP3 overexpression may promote cellular carcinogenesis.

The endometrium undergoes extensive remodeling during the pre-implantation period to establish endometrial receptivity in response to pregnancy signals [61,62]. The receptivity of endometrium can be partially explained by the distribution and functional capacity of the microvilli in endometrial epithelial cells [63]. Compared with day 0 of the estrous cycle, the number of microvilli in endometrium of dairy cows on the 14th day was significantly lower [64]. The reduction in microvilli in endometrial epithelial cells is mainly involved in the formation of pinopodes and promoting embryo adhesion. Integrins and OPN are potential mediators for blastocysts to attach to the endometrium and initiate implantation [65]. Bovine trophoblast cells have the characteristics of migration and fusion with uterine epithelial cells, and are composed of five α-subunits (ITGA2B, ITGA3, ITGA5, ITGA8 and ITGAV) and two β-subunits (ITGB1 and ITGB3) [66]. However, FABP3 overexpression had no significant effects on the adhesion of microvilli and trophoblast cell spheres in endometrial epithelial cells as well as the expression of ITGB3 and OPN, while it down-regulated the expression of ZO-1 and CDH1, suggesting that FABP3 may participate in regulating the dynamic remodeling of endometrium in dairy cows.

**Figure 8:**
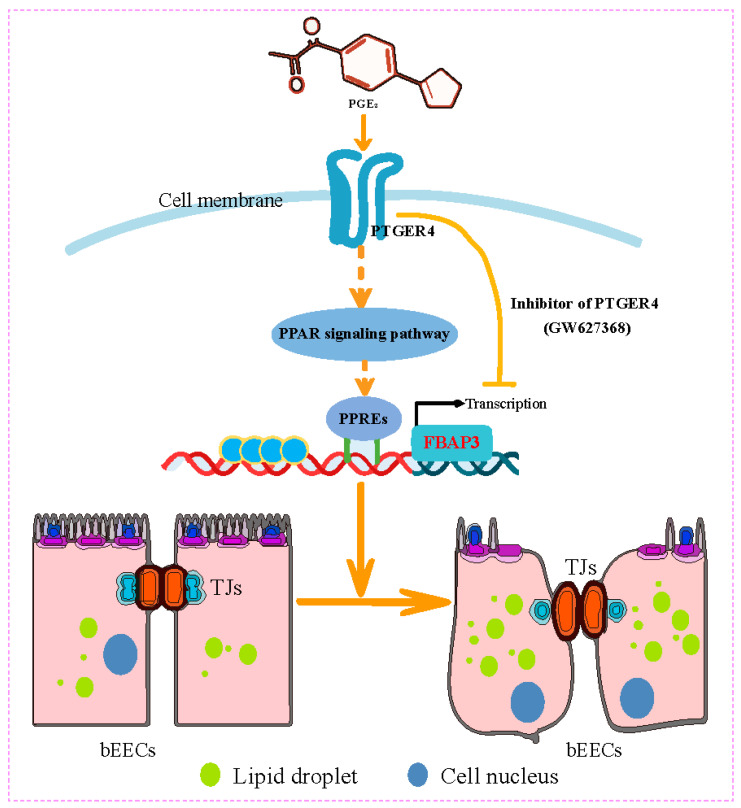
The mechanistic scheme of this study. A schematic model showing that FABP3 mediates lipid droplet accumulation and adhesive capacity in bovine endometrial epithelial cells via PGE_2_/PTGER4/PPAR axis. TJs: tight junctions; bEECs: bovine endometrial epithelial cells.

In ruminants, PGE_2_ is involved in multiple processes of early pregnancy, including conceptus elongation, maternal pregnancy recognition and embryo implantation. It mainly exerts multiple physiological functions by binding to different E-type prostaglandin receptors (PTGER1, PTGER2, PTGER3 and PTGER4) [67]. In this study, two types of receptors (PTGER2 and PTGER4) are mainly expressed in endometrial epithelial cells of dairy cows. This result is consistent with previous studies showing that the endometrium of cattle mainly expresses two receptors (PTGER2 and PTGER4), with temporal and cell-specific expression in the endometrium during the early stage of pregnancy or the estrus cycle [68]. Herein, PGE_2_ mainly regulates the expression of FABP3 in bEECs by binding to PTGER4, by interference with PTGER2, PTGER4, or pretreatment with PTGER4 inhibitor GW627368. However, the potential synergistic effect of PTGER2 cannot be ruled out. In previous studies, PTGER4 has been found to be a primary regulatory factor for PGE_2_ secretion and function [69,70]. In cattle, PTGER4 is more highly expressed in endometrial explants than PTGER2 [71], and its expression pattern is similar to that of PGE_2_ secretion [7]. Therefore, PTGER4 may be a primary receptor involved in PGE_2_-mediated signaling in endometrial cells. Moreover, FABP3 is elevated in the endometrial tissue of dairy cows during mid-diestrus compared to metestrus, with predominant localization in the luminal and superficial glandular epithelium. Collectively, this study demonstrates that FABP3 mediates lipid droplet accumulation and regulates the adhesive capacity of bovine endometrial epithelial cells through the PGE_2_/PTGER4/PPAR signaling axis (Figure 8). These results suggest that FABP3 serves as a functional link between lipid metabolic activity and implantation competence, and may represent a useful molecular indicator or target for improving reproductive performance in dairy cows. This study provides preliminary insights based on in vitro models, and further studies should aim to validate these findings in physiologically relevant systems, such as organ cultures or in vivo intervention experiments, which provide a more comprehensive understanding of hormonal regulation of lipid metabolism and cell morphology in reproduction.

## 5. Conclusions

In summary, this study identifies that FABP3 is a downstream effector of PGE_2_ signaling to mediate lipid droplet accumulation and regulate the adhesive capacity of bovine endometrial epithelial cells through the PGE_2_/PTGER4/PPAR signaling axis (Figure 8), which provides new insights into the metabolic regulation of endometrial receptivity.

## Data Availability

The original contributions presented in this study are included in the article/Supplementary Materials. Further inquiries can be directed to the corresponding authors.

## Supplementary Materials

The following supporting information can be downloaded at https://www.mdpi.com/article/10.3390/ani15233417/s1, Figure S1: The analysis of RNA-seq between P_4_ and P_4_ + PGE_2_ groups. Figure S2: The efficiency validation of FABP3 knockdown or overexpression in bEECs. Figure S3: The effect of cell remodeling in bEECs after FABP3 overexpression. Figure S4: The analysis of interference efficiency and treatment with inhibitors of PTGER2 and PTGER4. Table S1: Short hairpin interfering RNA (shRNA) inserts. Table S2: Primer pairs used for real-time quantitative PCR.
