# Supplementary material for: FABP3 Mediates Lipid Droplet Accumulation and Adhesive Capacity in Bovine Endometrial Epithelial Cells via PGE2/PTGER4/PPAR Axis
Source: Animals (Basel). 2025 Nov 26;15(23):3417. doi: 10.3390/ani15233417 (PMC12691251; doi:10.3390/ani15233417)
Supplement: Supplementary file 1 [file animals-15-03417-s001.zip › animals-3983488-supplementary.pdf]

Table S1. Short hairpin interfering RNA (shRNA) inserts.

shRNA	**Sequence (5’-3’)**
shFABP3-1 F	GATCCGGAAGTTAGTGGACAGCAACTCGAGTTGCTGTCCACTAACTTCCTTTTTG
shFABP3-1 R	AATTCAAAAAGGAAGTTAGTGGACAGCAACTCGAGTTGCTGTCCACTAACTTCCG
shFABP3-2 F	GATCCGCAGTTTGCACTCGTACTTCTCGAGAAGTACGAGTGCAAACTGCTTTTTG
shFABP3-2 R	AATTCAAAAAGCAGTTTGCACTCGTACTTCTCGAGAAGTACGAGTGCAAACTGCG
shFABP3-3 F	GATCCGCCTACCACAATCATCGAACTCGAGTTCGATGATTGTGGTAGGCTTTTTG
shFABP3-3 R	AATTCAAAAAGCCTACCACAATCATCGAACTCGAGTTCGATGATTGTGGTAGGCG
shPTGER2-1 F	GATCCACCACGTCCCCGGGTTGTACCTCGAGGTACAACCCGGGGACGTGGTTTTTG
shPTGER2-1 R	AATTCAAAAAACCACGTCCCCGGGTTGTACCTCGAGGTACAACCCGGGGACGTGGTG
shPTGER2-2 F	GATCCGTATGCGCAGAACCGGACTCCTCGAGGAGTCCGGTTCTGCGCATACTTTTTG
shPTGER2-2 R	AATTCAAAAAGTATGCGCAGAACCGGACTCCTCGAGGAGTCCGGTTCTGCGCATACG
shPTGER2-3 F	GATCCGAGCCACTGTGGCTCCCCTCCTCGAGGAGGGGAGCCACAGTGGCTCTTTTTG
shPTGER2-3 R	AATTCAAAAAGAGCCACTGTGGCTCCCCTCCTCGAGGAGGGGAGCCACAGTGGCTCG
shPTGER4-1 F	GATCCCCGAAGATGAACATCACCGCCTCGAGGCGGTGATGTTCATCTTCGGTTTTTG
shPTGER4-1 R	AATTCAAAAACCGAAGATGAACATCACCGCCTCGAGGCGGTGATGTTCATCTTCGGG
shPTGER4-2 F	GATCCGATGTTCATCTTCGGGGTCGCTCGAGCGACCCCGAAGATGAACATCTTTTTG
shPTGER4-2 R	AATTCAAAAAGATGTTCATCTTCGGGGTCGCTCGAGCGACCCCGAAGATGAACATCG
shPTGER4-3 F	GATCCCTACACACTGGTATGCGGGCCTCGAGGCCCGCATACCAGTGTGTAGTTTTTG
shPTGER4-3 R	AATTCAAAAACTACACACTGGTATGCGGGCCTCGAGGCCCGCATACCAGTGTGTAGG
shN F	GATCCTTCTCCGAACGTGTCACGTTTCAAGAGAACGTGACACGTTCGGAGAATTTTTG
shN R	AATTCAAAAAATTCTCCGAACGTGTCACGTTCTCTTGAAACGTGACACGTTCGGAGAAG
FABP3	F: CGGCTAGCTTCTCTGTCGTCTTTCCCAAC<br>R: CCCTCGAGTGCCTGTTTCTCGTAAGTACG

Table S2. Primer pairs used for real-time quantitative PCR.

Gene	Forward Primers (5’-3’)	Reverse Primers (5’-3’)
RPS9	CTGAAGCTGATCGGCGAGTA	GGGTCTTTCTCATCCAGCGT
PLIN2	AGTGAACTTGCCAGGAAGAATG	TTCATCTGTATCATCGTAGCCG
PTGER1	GGCCGCTGTTTTTGGCCGTG	CCTCCATGGCTGCCCTTGGC
PTGER2	TTTCCAGGGAAGGGTGTATG	GAGCATGAGTCAAGCCATGT
PTGER3	CAAGCCACATGAAGACCAGC	TCATTATCAGCAACGGCGAC
PTGER4	CGGTGATGTTCATCTTCGG	GTAGGCGTGGTTGATGGC
FABP3	CGTCTTTCCCAACCTAGCCC	TAGCAAAACCGACACCGAGT
ACSL4	GGGCCCCTCTTATTTGCTGT	TCACCAGTGCAAAACCCT
ACSL6	TTGCCAGATCCTGGGAGTTCT	GTCCCCTATTACGGATCGCC
EHHADH	ACAACTGTTGCCCGTGGAAT	TCTAATATCGGCACCTGCACA
DBI	CTGGAATGAGCTGAAAGGGAC	TGGCAGCCAAACTCAGTCTC

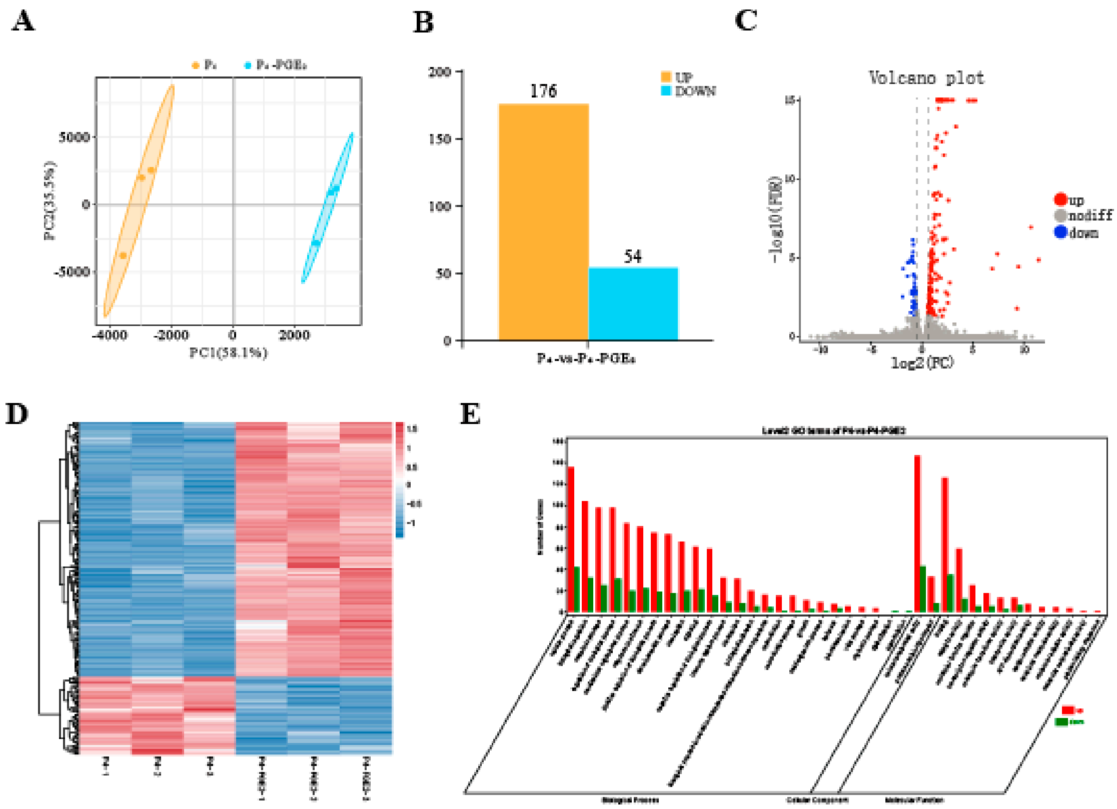

Figure S1. The analysis of RNA-seq between P_4 and P_4+PGE_2 groups. **A.** The analysis of PCA between P_4 and P_4+PGE_2 groups. **B.** The histogram of differentially expressed genes between P_4 and P_4+PGE_2 groups. **C.** The volcano plot of differentially expressed genes (DEGs) between P_4 and P_4+PGE_2 groups. **D.** The heatmap of DEGs between P_4 and P_4+PGE_2 groups. **E.** The GO analysis of DEGs between P_4 and P_4+PGE_2 groups.

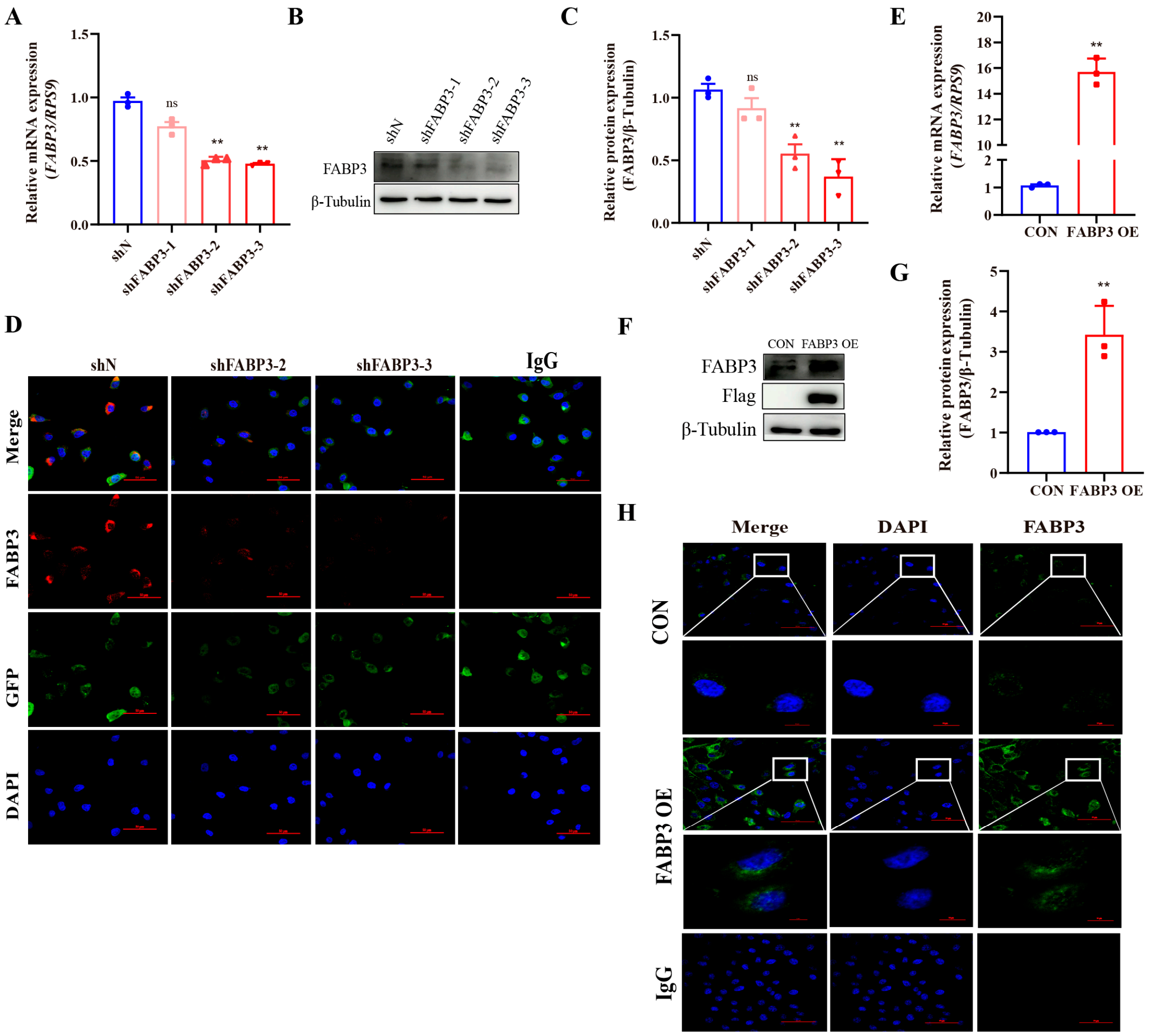

Figure S2. The efficiency validation of FABP3 knockdown or overexpression in bEECs. **A, E.** The analysis of *FABP3* expression in knockdown or overexpression cell lines, respectively. **B, C.** The representative images and analysis of FABP3 expression in knockdown cell lines, respectively. **F, G.** The representative images and analysis of FABP3 expression in overexpression cell lines, respectively. **D.** The representative images of FABP3 staining in knockdown cell lines. Scale bar: 50 μm; **H.** The representative images of FABP3 staining in overexpression cell lines. Scale bar: 50 μm (Others), 10 μm (Enlarge).

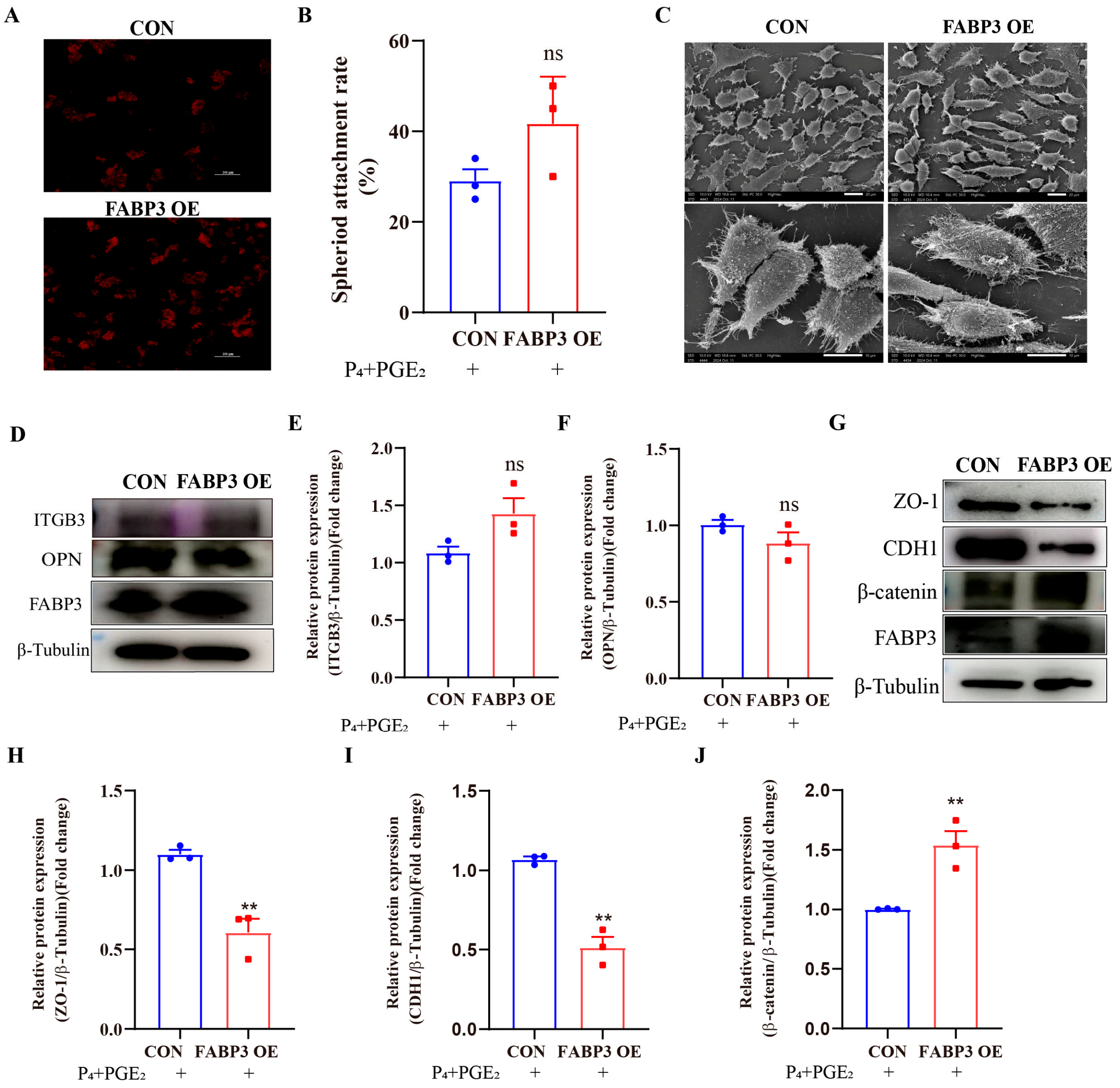

Figure S3. The effect of cell remodeling in bEECs after FABP3 overexpression. **A, B.** The representative images and analysis of BTCs spheroid adhesion rate in bEECs after FABP3 overexpression, respectively. BTCs: Bovine placental trophoblast cells. Scale bar: 200 μm **C.** The representative images of scanning electron microscopy in microvilli on the bEECs surface after FABP3 overexpression. Scale bar: 20 μm (Others), 10 μm (Enlarge). **D-F.** The representative images and analysis of the protein expression related to cell adhesion (ITGB3 and OPN) in bEECs between CON and FABP3 overexpression (FABP3 OE) groups, respectively. **G-J.** The representative images and analysis of the protein expression (ZO-1, CDH1 and β-catenin) in bEECs between CON and FABP3 overexpression (FABP3 OE) groups, respectively.

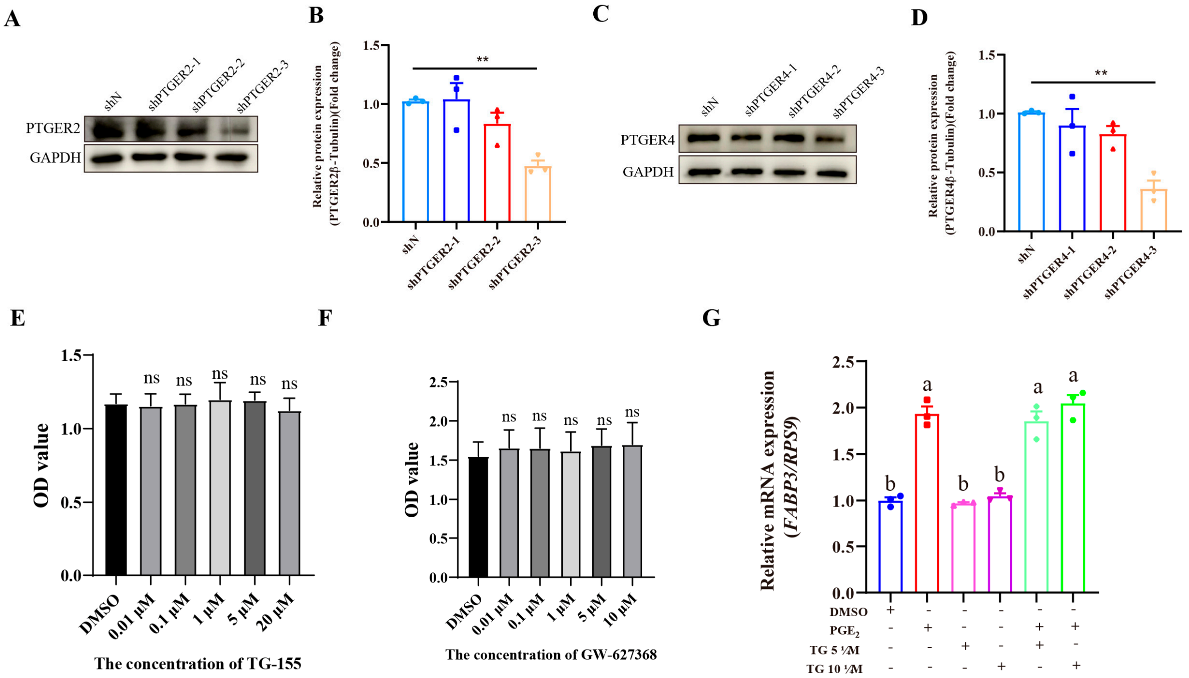

Figure S4. The analysis of interference efficiency and treatment with inhibitors of PTGER2 and PTGER4. **A, B.** The representative images and analysis of PTGER2 expression in bEECs after PTGER2 interference, respectively. **C, D.** The representative images and analysis of PTGER4 expression in bEECs after PTGER4 interference, respectively. **E, F.** The analysis of OD value in bEECs after inhibitor treatment of PTGER2 (TG-155) or PTGER4 (GW-627368), respectively. **G.** The analysis of *FABP3* expression in bEECs after treatment of PGE_2 and $PTGER_2$ inhibitor (TG-155).
